# Extensive population admixture on drone congregation areas of the giant honeybee, *Apis dorsata* (Fabricius, 1793)

**DOI:** 10.1002/ece3.1284

**Published:** 2014-12-02

**Authors:** Alexis L Beaurepaire, Bernard F Kraus, Gudrun Koeniger, Nikolaus Koeniger, Herbert Lim, Robin F A Moritz

**Affiliations:** 1 Institut für Biologie, Martin Luther Universität Halle-WittenbergHoher Weg 4, 06108, Halle (Saale), Germany; 2 Department of Laboratory Medicine, University Hospital HalleErnst Grube Str. 40, 06120, Halle (Saale), Germany; 3 Institut für Bienenkunde (Polytechnische Gesellschaft), Goethe Universität Frankfurt/MKarl-von-Frisch-Weg 2, 6347, Oberursel/Ts, Germany; 4 Agricultural Research Station TenomPeti Surat 197, Tenom, 89908, Sabah, Malaysia; 5 German Centre for Integrative Biodiversity Research (iDiv) Halle-Jena-LeipzigDeutscher Platz 5e, 04103, Leipzig, Germany

**Keywords:** *Apis dorsata*, drone congregation area, microsatellites, population genetics, sibship reconstruction analyses, spatiotemporal analyses

## Abstract

The giant honeybee *Apis dorsata* often forms dense colony aggregations which can include up to 200 often closely related nests in the same location, setting the stage for inbred matings. Yet, like in all other *Apis* species, *A. dorsata* queens mate in mid-air on lek like drone congregation areas (DCAs) where large numbers of males gather in flight. We here report how the drone composition of *A. dorsata* DCAs facilitates outbreeding, taking into the account both spatial (three DCAs) and temporal (subsequent sampling days) dynamics. We compared the drones’ genotypes at ten microsatellite DNA markers with those of the queen genotypes of six drone-producing colonies located close to the DCAs (Tenom, Sabah, Malaysia). None of 430 sampled drones originated from any of these nearby colonies. Moreover, we estimated that 141 unidentified colonies were contributing to the three DCAs. Most of these colonies were participating multiple times in the different locations and/or during the consecutive days of sampling. The drones sampled in the DCAs could be attributed to six subpopulations. These were all admixed in all DCA samples, increasing the effective population size an order of magnitude and preventing matings between potentially related queens and drones.

## Introduction

The aggregation of males for mating is a widespread phenomenon in the animal kingdom. It is typically characterized by the males of a given species clustering together, more or less densely, at specific points in time and space and thereby often forming so-called leks (Lloyd 1867). Lekking behavior of males has independently evolved in a large number of taxa and can be observed in animal groups as diverse as reptiles, amphibians, fish, birds, mammals, and insects. Because lekking is common in such a large number of very distant species, finding a single concise definition applying to all cases of male aggregations has been a great challenge. Höglund and Alatalo (1995) proposed a conciliatory definition, for which they stated that a lek is an “*aggregated male display that females attend primarily for the purpose of mating*”. On the basis of this definition, the male aggregations of honeybees (*Apis species*) called drone congregation areas (DCA, Ruttner and Ruttner 1968) match lekking behavior in many respects (Mueller et al. 2012). In this genus, sexually mature drones fly to well-defined areas outside their colonies to form DCAs on which up to thousands of males compete for mating with virgin queens. Although males do not display specific behaviors to mate, the competition involved during the race for pursuing the female also results in strong selection among the drones resulting in development of functional adaptations, as for instance a highly developed flight apparatus (Radloff et al. 2003) and huge compound eyes (Seidl 1980). Notably, these DCAs remain at the same location over many consecutive years (Ruttner and Ruttner 1968). Unlike the drones, which can only mate once and immediately die after copulation, honeybee queens mate with an exceptionally large number of drones (Moritz et al. 1995).

Many nonexclusive explanations concerning the origin and evolution of leks have been put forward, for example, group dilution effect on predation, clustering of individual due to habitat limitation or kin selection (Hoglund and Alatalo 1995; Kokko and Lindstrom 1996). In addition to these, the lek-based mating system of honeybees may have evolved for a different reason: inbreeding avoidance (Page 1980). In fact, minimizing the chances of mating among relatives are paramount for eusocial bees because of their complimentary sex determination system (Dzierzon 1845; de Camargo 1979). For these male-haploid species, inbreeding enhances the frequency of homozygosity at the complementary sex locus and results in the production of diploid males. These diploid males constitute a sex-linked genetic load (Zayed and Packer 2005), as they are sterile and do not participate in the colony tasks. In honeybees, such individuals are cannibalized by the workers at an early developmental stage (Woyke 1963). Diploid drones are produced instead of workers, which inhibits colony development and can severely reduce colony fitness (Hedrick et al. 2006).

In all honeybee species, the queens are extremely polyandrous (Tarpy et al. 2004). This characteristic further reduces the potential impact of inbreeding at the colony level. By mating with many drones, the queen diminishes the risks to lose her whole progeny if one of the males she mates with carries the same sex allele (Shakolsky 1976; Page 1980). The most extreme example of polyandry in honeybees is found in the giant honeybee *Apis dorsata,* in which queen's mate with an average of 44.2 ± 27.15 drones over up to six consecutive days (Moritz et al. 1995; Tan et al. 1999; Wattanachaiyingcharoen et al. 2003). *Apis dorsata* is native to the tropical forests of southeast Asia, where it builds single-comb nests in the open under thick tree branches or man-made structures. Colonies are headed by a single queen and can host as up to 50,000 individuals, including about 5000 drones and a few freshly emerged virgin queens (Tan 2007). Once they are mature, hundreds to thousands of giant honeybee males form DCAs shortly before dusk under the canopy of tall, emergent trees (Koeniger et al. 1994). In contrast to other *Apis* species, the daily mating period of *A. dorsata* is extremely short and drones fly only once a day for about 20–30 min (Koeniger and Wijayagunesekera 1976).

The life cycle of *A. dorsata* colonies includes phases of extensive migrations (Kahona et al. 1999). When the local environmental conditions degrade, and the food becomes scarce, entire colonies leave their comb to form migratory swarms (Itioka et al. 2001). Although these migratory swarms can travel up to 200 km (Koeniger and Koeniger 1980), they seem to return exactly to the same nesting tree in subsequent seasons (Neumann et al. 2000; Paar et al. 2000). Additionally, related *A. dorsata* queens tend to aggregate in the same bee tree which can include as many as 200 colonies (Paar et al. 2004; Rattanawannee et al. 2013). As a result, a strong substructuring of the giant honeybees population can be expected, setting the stage for inbreeding. The male aggregations on DCAs might play an important role to overcome this risk if they facilitate the gene flow among these colony aggregations. Indeed, Kraus et al. (2005) detected a high diversity of drone genotypes on an *A. dorsata* DCA on four consecutive days. However, in order to show that DCAs counter inbreeding not just the drones but also the local queens are important. In this study, we therefore determine the relatedness of drones caught on three giant honeybees’ DCAs together with the queens of colonies located in their direct vicinity. We analyzed the genetic structure of three *A. dorsata* DCAs both in time and in space to assess whether the male aggregations can facilitate random mating, thus counteracting the inbreeding potential resulting from the closely related queens in the colony aggregations.

## Material and Methods

### Location and sampling

**Figure 1:**
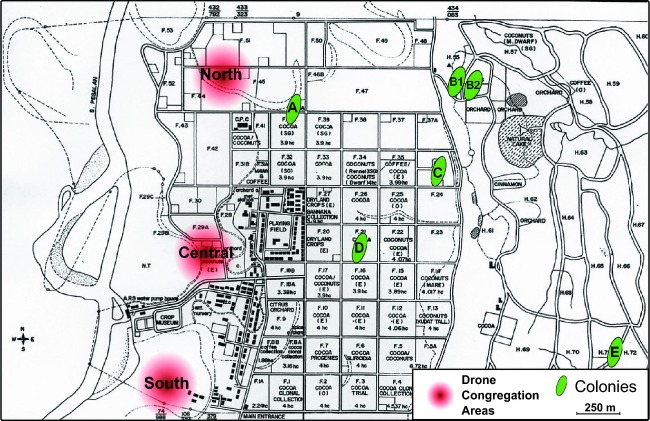
Schematic representation of the sampling site and subpopulation contribution to all three drone congregation areas (DCAs). Relative position of the sampled *Apis dorsata* resident colonies (red circles) and DCAs (green ellipses) in the Agricultural Research Station Lagud Sebrang of Tenom, Sabah, Malaysia.

A total of 430 drones were sampled between the 20.02.2005 and the 11.03.2005 on three distinct DCAs (called “North”, “Central”, and “South” hereafter) forming a line at the western edge of the Agricultural Research Station Lagud Sebrang of Tenom, Sabah, Malaysia (Fig. 1).

We caught *A. dorsata* drones at a height of 20–30 m at dusk under the canopy of large trees by lifting a large and heavy circular net (diameter 2.5 m) to a thick branch just over the center of the DCA. Under the net, a drone bait (1 mg 9-ODA) was tied to a nylon fishing line. As soon as a large group of drones was attracted to the bait, we let the net fall down and collected the drones caught inside on the ground (Koeniger et al. 2010).

During the sampling period, the agricultural station hosted twelve *A. dorsata* colonies. Out of these twelve colonies, 144 workers were sampled from six mature, drone-producing colonies located <2000 m east of the DCAs (Fig. 1). The other six colonies present in the study site had to be discarded as the number of sampled workers was too low and their queens’ genotypes could not unambiguously be estimated. As *A. dorsata* workers fiercely defend their colony, the sampling procedure was carried out during the night. A helium balloon with a piece of tape covered with insect glue was raised until the tape touched the colony. After getting the trap down, stuck workers could then be safely removed from the tape. All samples were stored in 70% ethanol directly after collection.

### DNA extraction and microsatellite typing

DNA was extracted from the hind leg of the 430 sampled drones (for each DCA: 96 the first day and 46 in North and 48 in Central and South, the second day as less drones were sampled) and 24 workers each from the six resident colonies with a routine Chelex protocol (Walsh et al. 1991). The samples were genotyped at ten unlinked microsatellite DNA loci (Solignac et al. 2003) using the Fragment Profiler software V. 1.2 of the MEGABACE DNA Analysis System independently by two scorers following standard procedures. When in disagreement, the results of the two scorers were typed a third time, and a consensus genotype was adopted.

### Analysis of the resident colonies

The genotype of the sampled colonies’ queens (“resident queens”) and drones (“resident drones”) were derived from the worker genotypes of all six colonies by Mendelian inference. The number of alleles and the allelic richness for the ten loci from the drones and worker genotypes were calculated using Fstat V2.9.3 (Goudet 1995). Furthermore, the difference of allele between the resident colony queens and resident colony drones was estimated for each colony.

### Analysis of the DCA composition

On the basis of the resident queen genotypes, we tested whether and how many of the sampled drones originated from the six neighboring colonies. This was done by comparing all sampled drone genotypes with the resident queen genotypes using a custom-made algorithm (Wolf et al. 2011) and the software COLONY 2.0.3.0 (Wang 2004) in parallel. For the latter software, appropriate individual marker error rates were added by comparing the results of the two independent genotypings.

We then used the software COLONY to estimate the number of nonresident colonies which had produced the remaining unassigned drones. This way, every drone which did not match a resident queen genotype could be assigned to a putative mother colony without prior queen information. Combining these two approaches, we finally obtained a total estimate of the number of colonies contributing drones to a given DCA location and day. Additionally, to estimate how accurately the sampling reflected the total number of colony present in the DCAs the nonsampling and nondetection error coefficients were calculated following Boomsma and Ratnieks (1996). To estimate whether the lower sampled size in the second day of sampling significantly increased the number of colony nonsampled, the nondetection error values between the 2 days of sampling were compared.

### Analysis of the contributing colonies

After identifying all the participating colonies, the overall contribution could be determined as drone number sent by each colony to the DCAs. This overall colony contribution was used to estimate whether the colonies contributed randomly to the DCAs. To do so, a goodness-of-fit test using the Chi-squared test statistics was used to compare the frequency of the observed colony contribution distribution to a distribution expected under a Poisson model. Additionally, the number of drones individual colonies sent to each DCA was estimated for the subsequent days.

### Analysis of the genetic structure

To infer the level of spatial genetic differentiation between the resident colonies and the different DCA locations, we calculated pairwise *F*_ST_ values using the software MSA v. 4.05 (Dieringer and Schlötterer 2003). To avoid the overrepresentation of siblings in the estimation, only one drone per inferred colony was kept in each DCA location and day of sampling. *F*_ST_ values were similarly calculated to infer temporal genetic differentiation between the 2 days of sampling within each DCA. For both temporal and spatial analyses, significant levels were estimated as the proportion of permuted *F*_ST_ values equal or greater than the observed *F*_ST_ after 10,000 allele permutation for each pair of populations and corrected after Bonferroni. Additionally, Jost's D estimator for genetic differentiation (Jost 2008) was calculated accordingly for each pair of DCA using the online version of the software SMOGD (Crawford 2010).

To estimate the number of subpopulations contributing to the DCAs and compare them to the drones originating from the resident colonies, the software Structure 2.3.4 (Pritchard et al. 2000) was utilized based on the sampled drones and derived resident drone genotypes (nonadmixture model; burn-in length: 100,000, run length: 250,000; 25 iterations from *K* = 1 to *K* = 8). From the results given by Structure, the delta *K* value (Evanno et al. 2005) was inferred using Structure Harvester (Earl and vonHoldt 2012). The software *CLUMPP* (Jakobsson and Rosenberg 2007) was used to find the optimal cluster membership alignment of our Structure analysis over the 25 iterations. These results were plotted using the software *Distruct* (Rosenberg 2004).

Finally, a Chi-squared test was performed to test the relationship between the inferred subpopulations and the DCA locations and days of sampling. To perform this test, observed proportion of drones per subpopulation in each DCA location and day of sampling were derived from the results of the optimal cluster membership given by the software *CLUMPP*. The value of the Chi-squared test was calculated manually using a contingency table (subpopulations × DCA location/days).

## Results

### Analysis of the resident colonies

A large number of alleles ranging from 8 to 62 over all ten microsatellite loci greatly facilitated the analyses (Table 1). The genotype of the queens and drones siring the six resident colonies could be unambiguously reconstructed from the worker genotypes. The resident queens had in average 4.60 ± 2.06 alleles in common out of 20 (two alleles for each of the 10 markers). Remarkably, the maximum number of alleles shared between two colonies (9) was between the colonies B1 and B2 which were located on the same tree (Appendix S2a). However, all resident queen genotypes differed for at least three of the ten markers used, indicating than none were related as mother–daughter or sister colonies. In average, the frequency of alleles shared between the resident drones and the resident queens coming from the same colony was 27% (Appendix S2b).

**Table 1:** Information about the microsatellite loci used.

Locus	All DCA units			Colonies		
	
	*N*_A_	*R*_t_	*H*_e_	*N*_A_	*R*_t_	*H*_e_
4978	20	14.98	0.872	16	15.58	0.848
6734	20	15.13	0.881	16	14.71	0.894
AC074	62	34.78	0.966	36	34.82	0.939
AT113	26	12.92	0.769	26	22.35	0.796
AT129	25	16.91	0.921	24	22.27	0.924
B124	18	8.28	0.631	10	9.80	0.651
BI012	11	6.16	0.577	8	7.72	0.561
BI098	57	34.11	0.955	46	42.31	0.970
BI226	20	12.65	0.789	14	14.00	0.803
BI234	19	13.39	0.853	12	11.88	0.636

Number of alleles and allelic richness scored in the DCAs and colonies at the 10 microsatellite marker used. *N*_A_, number of alleles; *R*_t_, allelic richness per locus overall samples; *H*_e_, expected heterozygosity; DCA, drone congregation areas; Left side: information on the overall DCAs; Right side: information for the resident colonies.

### Analysis of the DCA composition

None of the 430 drone genotypes matched any of the inferred six queen genotypes neither using the custom-made algorithm nor Colony 2.0.3.0. Over all DCAs, only an average of 2.36 ± 1.29 drones alleles (out of ten maximum) were matching correctly one of the queens’ genotype (Appendix S3). The subsequent analysis to assign the drones to inferred queens using again Colony 2.0.3.0 revealed that a total of 135 colonies contributed to the three DCAs over the whole sampling period with an overall average of 3.18 ± 1.90 drones per colony. The estimated nondetection error (two drones have the same allele combination at all loci) was very low (2 × 10^−9^), indicating that the number of marker used and their polymorphism permitted to characterize unambiguously and accurately different individuals. The estimated nonsampling error (colonies not sampled due to sample size) was as little as 5.58 colonies over all DCAs, illustrating that very few colonies contributing to the DCAs remained undetected. Furthermore, no significant differences between the nonsampling error between DCA North, Central, and South could be detected between Day I (12, 16 and 10, respectively) and Day II (9, 6, and 11, respectively; Fisher's test, *P* > 0.05).

### Analysis of the contributing colonies

**Figure 2:**
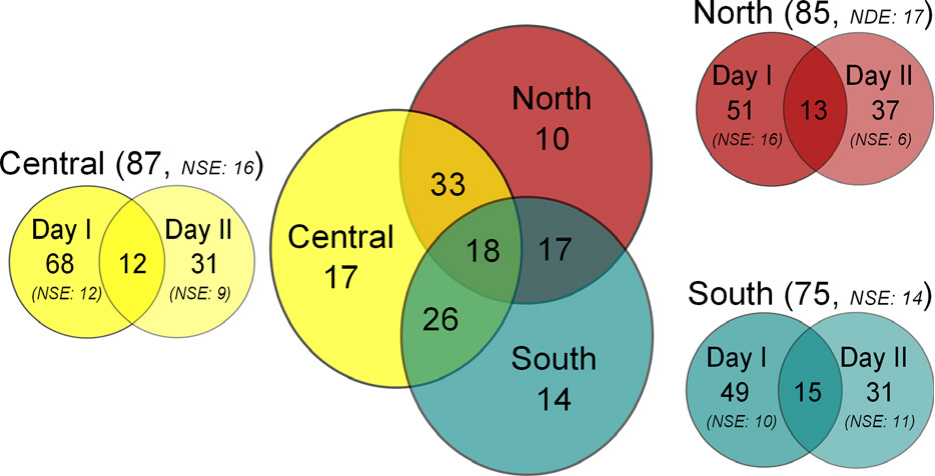
Amount of colonies contributing to the three drone congregation areas (DCA) locations. Venn diagram shows the number of colonies contributing to each DCA locations (North, Central, and South). The numbers indicate the amount of colonies contributing with at least one drone to the considered DCA location or multiple DCA locations. *NSE*: inferred nonsampling error.

The result of the goodness-of-fit test was highly significant (*P* < 0.001, *χ*^2^ = 25.94, df = 6), indicating that the distribution of the drones sent by the colonies did not follow a random distribution reflected by the Poisson model. About a third of the colonies were found on a single DCA only (30.37%), the majority was detected in two (56.30%) and only few in all three DCAs (13.33%; Fig. 2). Most colonies (≥80%) were found in only one of the two sampling days for the three DCAs (Fig. 2).

### Analysis of the overall genetic structure

Low but significant levels of genetic differentiation were obtained between the resident colonies and the three DCAs, but not among the DCA locations (Table 2A). The same test revealed no significant genetic differentiation between the 2 days of sampling (Table 2B). The low absolute *F*_ST_ values are an inevitable result of the highly polymorphic markers (Whitlock 2011) unlike Jost's D estimator which values are all an order of magnitude higher, indicating low**-**to**-**moderate population differentiation for all comparisons (Table 2A and B).

**Table 2:** Results of the pairwise tests for population differentiation.

	Colonies	North		Central		South	
			
		*F*_ST_	D	*F*_ST_	D	*F*_ST_	D
A. Spatial pairwise genetic differentiation							
Colonies		**0.024**	0.138	**0.030**	0.148	**0.029**	0.189
North	***			0.002	0.037	0.000	0.021
Central	***	N.S.				0.000	0.022
South	***	N.S.		N.S.			

DCA	*F*_ST_	*P*-values	D
B. Temporal pairwise genetic differentiation			
North	0.006	N.S.	0.078
Central	0.011	N.S.	0.077
South	0.012	N.S.	0.075

Results of the pairwise population differentiation test between the resident colonies and the DCAs and within DCAs (spatial and temporal differentiation). *F*_ST_ values and level of significance calculated for 10,000 permutation of alleles (N.S., nonsignificant after Bonferroni's correction; ***P-values < 0.001); D, Jost's D estimator value; DCA, drone congregation areas; A: Spatial comparison between DCA location and the resident colonies. On the right-upper part: ***F*_ST_ values**, on the left-lower part: associated *P*-values; B: comparison between 2 days of sampling within each DCAs.

**Figure 3:**
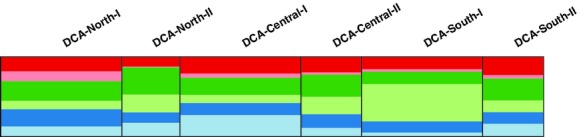
Results from structure analysis between drone congregation areas (DCA) units. Result of the optimal cluster membership alignment of the Structure software analysis over the 25 iterations between the different DCA locations and days of sampling. The *Y* axis indicates the probability of a population membership to one of the six estimated *K* subpopulations, each represented by a distinct color (in green: subpopulation 1, in orange: subpopulation 6). From left to right: the pooled drones from different DCA location (North, Central, and South, respectively) and 2 days of sampling (I and II).

The most likely number of subpopulations inferred by the Structure software was *K* = 6 (Mean LnP(*K* = 6) = −10708.10), which was further confirmed by Evanno's delta *K* estimator (Δ_*k* = 6_ = 199.02). All inferred subpopulations were found in all DCA locations and days of sampling, each of them being represented by an average of 22.50 ± 3.06 colonies over all the DCAs (Fig. 3). The individual optimal cluster membership alignment for each of the resident colonies and DCA locations and days of sampling is shown in Appendix S1.

The statistical analyses based on the six subpopulations revealed highly significant differences between the observed contribution of the subpopulations compared with a random contribution (*P* < 0.001, χ^2^ = 83.87, df = 25). These differences are primarily due to the overrepresentation of a few subpopulations at some of the DCA units (subpopulation 5 in DCA North I, subpopulation 1 in DCA Central I, and subpopulation 3 in DCA South I).

## Discussion

The main result of this study is that not a single one of the 430 genotyped drones originated from any of the six resident colonies next to the DCAs. Instead, we estimated that 135 unidentified colonies contributed to the sampled DCAs. We sampled on average 3.18 ± 1.90 drones per colony and estimated only ten of these colonies to be represented by a single drone. Accordingly, the nondetection and nonsampling errors were very low, indicating that the sampling covered the vast majority of colonies participating in the DCAs. Although we found that fewer colonies were contributing to the second day of sampling, the nonsampling errors were similar between both days. This result indicates that the lower sample size for the second day may not have affected our estimates. Moreover, our results show that although we could identify six distinct subpopulations that contributed to the giant honeybees DCAs, their drones were efficiently mixed on all DCAs into one fused allelic pool.

The comparison of alleles between the different resident colonies indicated that none could be related as sister colonies. However, two colonies (B1 and B2) located in the same tree showed more similarities than with the rest of the resident colonies (Appendix S2a). This result can be due to the tendency for related colonies to aggregate previously described in *A. dorsata* (Paar et al. 2004).

In the genus *Apis*, the only related record based on colony contribution to DCAs reported that in the Western honeybee *Apis mellifera* drones tend to fly to the nearer DCAs (Koeniger et al. 2005). Similarly, short mating flight distances were also reported by following the hum of drones of *A. dorsata* by Koeniger et al. (1994) in the exact same sampling location as this study. Unlike these observations*,* we found no drones from six neighboring colonies in the giant honeybees DCAs we analyzed. As we only genotyped six of the 12 resident colonies, we cannot exclude that the other six colonies located in the sampling site were not contributing to the DCAs. Nevertheless, we could show that the most closely located colonies did not contribute to the sampled DCAs. Even if the six remaining colonies had contributed, they were vastly outnumbered by the other 129 colonies which can be excluded to be resident colonies. Our findings are very similar to reports of male aggregation in some other eusocial bee species. For example, Mueller et al. (2012) showed for male aggregations of *Scaptotrigona mexicana* that none of the males in an aggregation originated from neighboring colonies located at the same site. In this species, populations are strongly substructured as daughter colonies do not disperse far away from their mother colony, and inbreeding may affect the colonies even more than in honeybees as queens are only singly mated. Although *A. dorsata* queens are highly polyandrous, and subpopulations are not as structured as for *S. mexicana*, we could not detect local drones on the DCA. This lack of local drones suggests that reproduction in the giant honeybees is not just random, but that the drones’ mating flight behavior might prevent matings with related queens.

The agricultural station where the sampling was conducted is about 8 km² in size and surveys taking place for decades in this area indicated that the site housed twelve *A. dorsata* colonies during the time of sampling. Using the colony density on the site (about 1.50 colonies/km²) as an estimator for overall colony density in the region, the 135 colonies we sampled would have come from a circular area covering as much as 94 km² resulting in a flight radius of 5.47 km around the DCAs. This is more than the range reported for *A. mellifera* drones (Ruttner 1966) and previous reports on *A. dorsata* DCAs locations (Koeniger et al. 1994). As the colony density on the agricultural station is likely to be lower than the actual density on the wild areas surrounding the sampling site, this absolute flight radius is probably a conservative estimate. As *A. dorsata* drones are particularly capable of flying fast over long distance (Radloff et al. 2003), the mating range appears fully realistic given the durations of drone mating flights (Koeniger et al. 1994): if the giant honeybee drones fly at the same speed as workers (about 7 m/sec, Dyer and Seeley 1987), it would take them 13 min at most to reach the DCA. This estimation would fit well within the daily drone flight duration of about 20–30 min in *A. dorsata* (Koeniger et al. 1994; Tan et al. 1999; Otis et al. 2000).

We found that the estimated colonies did neither contribute their drones randomly nor equally to the sampled DCAs. The various inferred subpopulations were not evenly represented on the DCAs (Fig. 3). These results are due to the differential representation of the sampled colonies, which might be due to a suite of factors that remained uncontrolled; including colony proximity to the DCA, number of drones produced, and wind direction that might interfere with the number of drones at a given DCA. Most of the 135 colonies we sampled once on a given DCA could be resampled on other DCAs, indicating that dispersion already occurs among the drones at the colony level.

We found no significant genetic differentiation among all DCA sample sets. The very low values for both *F*_ST_ and Jost's D estimates for the spatial comparison reflect the fact that most colonies could be resampled at different locations. The slightly higher and not significantly different estimates for the temporal differentiation may be due to the small number of colonies on the two consecutive days of sampling. In contrast, we found a significant genetic differentiation between the drone population that had sired the workers of the resident colonies and those caught on the DCAs. This may not be surprising as these two groups of drones belong to two different generations after seasonal migrations of the colonies.

Our findings corroborate the reports of Kraus et al. (2005) who showed that DCAs can be places of high promiscuity, fusing multiple subpopulations on one site. This has profound consequences on the genetic effective size (*N*_e_) of *A. dorsata* populations. On the basis of the workers sampled in this study, we could estimate the average mating frequency of the six resident queens to be 13.5 ± 2.41. Yet, based on 24 workers only, this measure is very likely to be biased. More robust sample sizes yielded an average effective paternity of 44.2 males per queen for *A. dorsata* (Tarpy et al. 2004). Given this latter estimate, the overall effective population size if only the twelve colonies found within the agricultural station would be considered is *N*_e(resident colonies)_ = 26.7. This is much less compared with the average *N*_e_ for each DCA location and day of sampling, *N*_e(average DCAs)_ = 130.12 ± 6.25. As *A. dorsata* virgin queens can visit different DCAs during their mating flights and perform several mating flights on consecutive days (Tan et al. 1999), they may encounter even larger effective populations. The overall effective population size across the three closely located DCAs and the two consecutive days of sampling yielded an effective size of *N*_e(overall)_ = 314. This latter estimate may still be an extremely conservative estimate of the diversity of drones’ genotypes queens actually encounter by mating on the leklike DCAs if she simultaneously visits more distant DCAs than we sampled. The effective population size found in *A. dorsata* DCAs is enhanced by more than an order of magnitude in comparison with the local resident colonies. This extreme admixture of drones from various subpopulations is most suited to drastically reduce the probability for queens to mate with potentially related drones.

Irrespective of the evolutionary drivers of this extraordinary mating behavior, the DCAs seem to meet most of the requirements to be qualified as a lek as defined by Bradbury (1981) who proposed four main criteria to distinguish it: (1) males provide no parental care; (2) males are aggregated in arenas to which females come to mate; (3) the display arena contains no significant resources for females; and (4) females have an opportunity to chose their mates. In honeybees, drones do not provide brood care as reproduction causes their death and they group in well-defined areas for the only purpose of mating. Additionally, no significant resources are provided to queens when they fly to a DCA. Queens seem to not actively choose their mating partners as they are chased by comets of drones which compete for mating. However, although the queen does not fly to a DCA to mate with the fittest male on the lek but rather to avoid mating with related drones, the functional role is very similar; maximizing the potential fitness of the offspring. The honeybee queen does not achieve this by a selective mating but rather by obtaining a set of most diverse matings. So, if maximizing fitness for females is the purpose of the lek, DCAs are clearly matching this definition and will facilitate comparisons to other lek-based mating systems (Mueller et al. 2012).

In honeybees, the DCAs provide a mating strategy for queens to easily facilitate inbreeding avoidance, which is considered as one of the major drivers of the evolution of this lekking mating system (Page 1980). When defining the DCA of *Apis* as a lek, it has therefore very different consequences compared with other taxa. Typically, females choose their mating partner on leks according to certain taxa-specific phenotypic criteria. If these traits are heritable, this will ultimately lead to the depletion of genetic variation due to strong sexual selection. This theory is known as the lek paradox (Borgia 1979) and has become a topic of numerous debates. Clearly, in the honeybee, the opposite is achieved. It seems the queen seeks to mate with as many males as possible thereby averaging over phenotypes but reducing the impact of inbreeding at the colony level (Page 1980).

To conclude, we could demonstrate that the mixing of the giant honeybee gene pool is achieved in multiple ways: extreme polyandry of the queen and excessive mixing of populations by drone mating flights.

## Data Accessibility

Doi: 10.5061/dryad.64jp5.

## Supporting Information

**Appendix S1.** Results from structure analysis at the individual level. **Appendix S2.** Frequency of alleles shared between and within the resident colonies. **Appendix S3.** Frequency of drones sharing resident queens’ alleles.
