# Supplementary material for: Extensive population admixture on drone congregation areas of the giant honeybee, Apis dorsata (Fabricius, 1793)
Source: Ecol Evol. 2014 Dec 2;4(24):4669–77. doi: 10.1002/ece3.1284 (PMC4278819; doi:10.1002/ece3.1284)
Supplement: Supplementary file 1 [file ece30004-4669-sd1.doc]

Supplementary Material

Appendix 1 – Results from Structure Analysis at the individual level

Result of the optimal individual membership alignment of the Structure software analysis over the 25 iterations between the different DCA units. The Y axis indicates the probability for each individual to belong to one of the six estimated K subpopulations, each represented by a distinct colour. From left to right: individual drones estimated from the six resident colonies and different DCA location (North, Central and South) and two days of sampling (I and II).

| 2.a. | B1 | B2 | C | D | E |
| --- | --- | --- | --- | --- | --- |
| A | 0.30 | 0.30 | 0.15 | 0.05 | 0.17 |
| B1 |  | 0.45 | 0.20 | 0.35 | 0.17 |
| B2 |  |  | 0.35 | 0.20 | 0.22 |
| C |  |  |  | 0.20 | 0.17 |
| D |  |  |  |  | 0.28 |

| 2.b. | A | B1 | B2 | C | D | E |
| --- | --- | --- | --- | --- | --- | --- |
| Avg | 0.26 | 0.33 | 0.31 | 0.20 | 0.23 | 0.29 |
| Stdev | 0.19 | 0.20 | 0.17 | 0.15 | 0.12 | 0.18 |

Appendix 2 – Frequency of alleles shared between and within the resident colonies

Tables indicating the frequency of alleles shared between each pair of resident colony queens (2.a.) and between each inferred resident drones in relation to the resident queen it mated with (2.b.). Avg: colony average over the different resident drones, Stdev: standard deviation relative to this average

Appendix 3 – Frequency of drones sharing resident queens' alleles

Graph representing the number of alleles shared between the six colonies and the percentage of the 430 sampled drones sharing alleles with a given resident colony queen, represented by different colours. In X-axis: number of alleles, in Y-axis: percentage of drones (over the 430 samples) sharing alleles with the considered colony.
